# Consensus Statement from the Society of Gynecologic Oncology of Canada on Folate Receptor α Testing in Ovarian Cancer

**DOI:** 10.3390/curroncol33060330

**Published:** 2026-06-01

**Authors:** Kim Ma, Basile Tessier-Cloutier, Alon D. Altman, Mark S. Carey, Josee-Lyne Ethier, Susie Lau, Cheng-Han Lee, Laura Hopkins, Katharina Kieser, Aalok Kumar, Shuk On Annie Leung, Julie M. V. Nguyen, Helen MacKay, Jacob McGee, Lina Salman, Shannon Salvador, Cidalia Sluce, Luiza Tatar, Alicia A. Tone, Anna Tinker, Elizabeth Tremblay, Ana C. Veneziani, Danielle Vicus, Stephen Welch, Sharon Windsor Harker, Melica Nourmoussavi Brodeur

**Affiliations:** 1 Division of Gynecologic Oncology, Jewish General Hospital, McGill University, Montréal, QC H3T 1E2, Canada; 2 Faculty of Medicine and Health Sciences, McGill University, Montréal, QC H4A 3J1, Canada; 3 Max Rady College of Medicine, Obstetrics, Gynecology and Reproductive Sciences, WN50014—Women’s Hospital, University of Manitoba, Winnipeg, MB R3A 1R9, Canada; aaltman@cancercare.mb.ca; 4 Division of Gynecologic Oncology, Vancouver Coastal Health; Department of Obstetrics and Gynecology, Faculty of Medicine, University of British Columbia, Vancouver, BC V6H 3N1, Canada; mark.carey@vch.ca (M.S.C.);; 5 Department of Medicine, Sunnybrook Odette Cancer Centre, University of Toronto, Toronto, ON M4N 3M5, Canada; 6 Segal Cancer Centre, Sir Mortimer B. Davis Institute of Medical Research, McGill University, Montréal, QC H3T 1E2, Canada; 7 Department of Laboratory Medicine and Pathology, University of Alberta, Edmonton, AB T6G 1C9, Canada; 8 Division of Gynecologic Oncology, Saskatoon Cancer Centre, Saskatoon, SK S7N 5B8, Canada; 9 Division of Gynaecologic Oncology, Dalhousie University, Halifax, NS B3H 1V7, Canada; kkieser@dal.ca; 10 BC Cancer-Surrey, University of British Columbia, Vancouver, BC V3V 1Z2, Canada; 11 McGill University Health Centre, Montreal, QC H4A 3J1, Canada; annie.leung@mcgill.ca (S.O.A.L.);; 12 Division of Gynecologic Oncology, Department of Obstetrics and Gynecology, McMaster University, Hamilton, ON L8S 4K1, Canada; 13 Department of Obstetrics and Gynecology, London Health Sciences Centre and Schulich School of Medicine and Dentistry, Western University, London, ON N6A 5W9, Canada; 14 Society of Gynecologic Oncology of Canada, Ottawa, ON K1H 8K3, Canada; 15 Ovarian Cancer Canada, Toronto, ON M2P 2A9, Canada; atone@ovariancanada.org; 16 Division of Gynecologic Oncology, Centre Hospitalier de l’Université de Montréal (CHUM), Montreal, QC H2X 0A9, Canada; 17 Département d’obstétrique et gynécologie, Université de Montréal, Montréal, QC H3T 1J4, Canada; 18 Department of Medical Oncology and Hematology, Princess Margaret Cancer Centre, University Health Network, Toronto, ON M5G 2C4, Canada; ana.veneziani@uhn.ca; 19 Division of Gynecologic Oncology, Department of Gynecology and Obstetrics, University of Toronto, Toronto, ON M5G 1E2, Canada; 20 Windsor Medical Communications, Inc., Hamilton, ON L8N 1Z6, Canada; sharon@windsormedical.ca

**Keywords:** epithelial ovarian cancer, folate receptor alpha, mirvetuximab soravtansine, consensus statement, companion diagnostics

## Abstract

Background: Folate receptor alpha (FRα), commonly expressed in epithelial ovarian cancers, is a clinically actionable biomarker following approval of the antibody–drug conjugate mirvetuximab soravtansine (MIRV). Pivotal trials showed that high FRα expression predicts MIRV benefit, creating the need for standardized testing to ensure timely, equitable access. Methods: To address the need for guidance on FRα testing, the Society of Gynecologic Oncology of Canada convened a multidisciplinary Expert Panel to review the evidence and integrate Canadian clinical, pathology, laboratory, and patient perspectives. Consensus recommendations were developed through structured evidence review, expert discussion, iterative revision, and patient partner input. Results: The panel issued recommendations on the clinical role and timing of FRα testing, tissue requirements, assay selection and validation, interpretation and reporting standards, laboratory quality assurance, reimbursement, and equitable access. It is recommended that FRα testing be available to all patients with epithelial ovarian cancer, with results available no later than platinum-resistant disease, using a validated assay, preferably the Ventana FOLR1 RxDx assay or an appropriately validated laboratory-developed test. Standardized synoptic reporting, participation in external quality assurance programs, and clear patient communication were deemed essential. Conclusions: These recommendations aim to promote integrated, equitable, standardized FRα testing across Canada and support timely identification of patients eligible for FRα-directed therapy, clinical trial enrollment, and future biomarker-driven treatment strategies.

## Simple Summary

Folate receptor alpha (FRα) is a protein that is found in large amounts on the surface of most epithelial ovarian cancer cells. FRα has become an important target for treatment by specifically seeking out and binding to cancer cells. Clinical trials for a recently approved medication, mirvetuximab soravtansine, demonstrate that if a patient’s cancer has high levels of FRα, they are likely to benefit from this targeted drug, offering a new treatment option for a disease with the lowest survival rate among gynecological cancers in Canada. This consensus statement recommends that all Canadians with epithelial ovarian cancer should have timely access to FRα testing to guide therapeutic decisions and provide access to life-prolonging medication.

## 1. Introduction

Epithelial ovarian cancer (EOC) remains the most lethal gynecologic malignancy worldwide, representing 90% of ovarian cancer cases and accounting for more than 300,000 new cases and over 200,000 deaths annually [1]. In Canada, the five-year survival rate for advanced-stage EOC remains below 50% despite surgical advances and the introduction of poly-(ADP-ribose)-polymerase (PARP) inhibitors, with most patients presenting in late–advanced stage [1,2,3].

Folate receptor α (FRα), encoded by the FOLR1 gene, is a glycosylphosphatidylinositol (GPI)-anchored protein that mediates folate transport into cells through receptor-mediated endocytosis [4,5]. FRα has minimal expression in most adult tissues; however, it is highly expressed in a wide range of epithelial malignancies, including non-small-cell lung cancer, endometrial cancer, and breast cancer, but its most consistent and clinically significant role is in EOC [6,7,8,9,10]. The restricted normal distribution and high prevalence in EOC tumours provide FRα with a uniquely favourable therapeutic index [1,3,11].

FRα expression has been documented in 76–89% of high-grade serous carcinoma [9,10,12], ~40% of low-grade serous carcinomas [12], 20–35% of endometrioid carcinomas [10,13], and 15–25% of clear cell carcinomas [10,13]. Mucinous ovarian carcinomas rarely express FRα (<10%) [10,13]. Importantly, FRα expression appears to be relatively stable between primary and recurrent tumours, allowing for the use of archival tissue in biomarker testing at relapse without the need for repeated biopsies in most cases [14].

### 1.1. Therapeutic Rationale

The centrality of FRα as a therapeutic biomarker is underscored by the development of mirvetuximab soravtansine (MIRV), an antibody–drug conjugate consisting of a humanized anti-FRα antibody linked to the maytansinoid DM4 toxin [8,12,15]. In the phase III MIRASOL trial, MIRV demonstrated superior progression-free survival and overall survival compared to investigator’s choice chemotherapy in FRα-positive platinum-resistant ovarian cancer [8,15,16,17]. These results led to accelerated approval by the U.S. Food and Drug Administration (FDA) in 2022, followed by regular FDA approval in March 2024, as well as Health Canada approval in 2025, establishing FRα testing as a clinically actionable biomarker [18,19].

Beyond MIRV, several investigational therapeutics are advancing the FRα field: STRO-002 (luveltamab tazevibulin), a next-generation antibody–drug conjugate, demonstrated durable responses in early-phase studies (NCT03748186) [20,21]; MORAb-202, a farletuzumab-eribulin ADC, has shown bystander killing and promising antitumor activity (NCT05613088) [13,22,23]; pre-clinical bispecific T-cell engagers (BiTEs), such as TNB-928B, exploit FRα selectivity to engage CD3-positive T cells against tumour targets [24,25]; and chimeric antigen receptor T-cell (CAR-T) therapies targeting FRα (e.g., MOv19-BBζ, NCT03585764) are in early-phase trials, although challenges remain due to potential on-target, off-tumour effects [26,27]. Intraoperative imaging with pafolacianine (Cytalux), a folate analog conjugated to a near-infrared dye, was FDA-approved in 2021 for enhanced visualization of ovarian tumours during cytoreductive surgery [27,28,29]. Together, these developments reinforce the need for consistent, validated FRα testing strategies across clinical settings.

### 1.2. Rationale for Consensus

Despite clinical trial success and regulatory approvals, FRα testing remains inconsistently implemented across Canadian institutions. Regional differences in biomarker funding and the laboratory biomarker testing landscape risk inequitable access to FRα-targeted therapies. Lessons from BRCA1/2 and homologous recombination deficiency (HRD) testing have demonstrated the challenges with inconsistent or delayed uptake [30]. Without harmonized national standards, eligible Canadian patients may face barriers in accessing FRα-targeted therapies such as MIRV depending on testing availability and accessibility, with delayed or missed treatment opportunities [30].

Therefore, this consensus statement was developed by a multidisciplinary panel of Canadian gynecologic oncologists, pathologists, medical oncologists, and patient partners to (1) define the role of FRα as a biomarker in EOC; (2) provide guidance on timing of testing and reflex strategies; (3) recommend validated testing methodologies and thresholds; (4) standardize reporting and quality assurance; and (5) ensure equitable national access through harmonized reimbursement.

## 2. Materials and Methods

### 2.1. Consensus Development Process

The Society of Gynecologic Oncology of Canada (GOC) established a Community of Practice for Biomarkers in Gynecological Cancer, who designated a multidisciplinary Expert Panel to develop national consensus recommendations for folate receptor α (FRα) testing in ovarian cancer. The methodology was modelled on established Canadian consensus processes for biomarker implementation and followed a structured, iterative approach designed to ensure clinical relevance, pan-Canadian representation, and methodological transparency.

### 2.2. Expert Panel Composition

Panel members were selected based on recognized expertise in gynecologic oncology, pathology, molecular diagnostics, and biomarker implementation. The initial working group comprised gynecologic oncologists (13), medical oncologists (5), gynecologic pathologists (2) from multiple provinces (West 6, ON 7, QC 7, East 1) and the Ovarian Cancer Canada scientific advisor to ensure regional representation and applicability across Canadian practice settings. All members were actively engaged in ovarian cancer care and/or biomarker testing programs and considered national opinion leaders in their respective fields.

### 2.3. Scope Definition and Methodological Framework

The Biomarker Community of Practice leaders met with members of the GOC board to define the scope of the project, identify key clinical questions, and agree on the consensus development methodology. The objective was to generate practical, implementation-focused recommendations for FRα testing in EOC in Canada, including indications for testing, timing of testing, specimen requirements, assay selection, reporting standards, quality assurance, reimbursement, and equitable access.

Given the rapidly evolving evidence base and the implementation-focused nature of the topic, the panel used a structured narrative evidence review combined with expert consensus interpretation, rather than a formal systematic review or GRADE-based guideline process. This approach was selected to allow integration of clinical trial data, regulatory documents, pathology assay validation data, Canadian laboratory practice considerations, and patient partner input. Recommendations were therefore interpreted as consensus-based guidance rather than formal evidence-graded clinical practice guidelines.

### 2.4. Evidence Review

A structured narrative literature review was conducted by K.M. and M.B. to synthesize evidence supporting FRα as a predictive biomarker, the performance of available FRα assays, and clinical outcomes associated with FRα-targeted therapies. Searches were performed in PubMed/MEDLINE and supplemented by review of regulatory documents, product monographs, companion diagnostic materials, clinical trial records, conference abstracts where relevant, and key publications known to panel members.

Search terms included combinations of: “folate receptor alpha,” “FRα,” “FOLR1,” “ovarian cancer,” “epithelial ovarian cancer,” “platinum-resistant ovarian cancer,” “mirvetuximab soravtansine,” “SORAYA,” “MIRASOL,” “VENTANA FOLR1,” “FOLR1 RxDx,” “immunohistochemistry,” “companion diagnostic,” “biomarker testing,” “assay validation,” “external quality assurance,” and “molecular testing access.” Searches focused on English-language publications and regulatory or technical documents available up to December 2025.

Evidence was prioritized according to clinical relevance. Highest priority was given to prospective clinical trials evaluating FRα-directed therapy, studies validating FRα immunohistochemistry assays, regulatory and companion diagnostic documents, and pathology quality assurance data. Additional evidence included observational studies of FRα expression by histologic subtype, studies of biomarker implementation in ovarian cancer and other malignancies, and literature addressing disparities in access to molecular testing. Evidence from established biomarker programs, including HER2, BRCA/HRD, PD-L1, and MMR/MSI, was reviewed to inform implementation, reporting, quality assurance, and equity recommendations in the Canadian context.

Formal risk-of-bias assessment and GRADE certainty ratings were not performed. Instead, the strength of each recommendation was considered qualitatively by the panel based on the consistency and directness of the evidence, clinical actionability, feasibility of implementation, patient impact, and relevance to the Canadian health system.

### 2.5. Drafting of Preliminary Recommendations

Following the evidence review, a steering subgroup composed of gynecologic oncology, medical oncology, and pathology representatives drafted preliminary recommendation statements. These statements addressed: (1) clinical indications and timing of FRα testing; (2) pre-analytical specimen considerations; (3) assay selection and validation; (4) pathology reporting; (5) quality assurance; and (6) patient communication and equitable access.

Each draft statement was accompanied by a brief evidence summary and rationale. Recommendations supported directly by prospective clinical trial evidence or validated companion diagnostic data were distinguished from recommendations based primarily on implementation considerations, expert opinion, or extrapolation from other biomarker programs.

### 2.6. Consensus Meeting and Iterative Revision

Draft recommendations were circulated to the full panel before the consensus meeting. A half-day in-person consensus meeting was then held to review and refine each recommendation. Panel members discussed the evidence base, feasibility of implementation, expected clinical impact, laboratory requirements, and applicability across Canadian provinces and practice settings.

Consensus was defined a priori as agreement by at least 80% of participating panel members. Where disagreement existed, recommendations were revised through structured discussion until the wording reflected the strongest statement acceptable to the group. Statements that did not meet the pre-specified threshold were modified until consensus was achieved. Final revised recommendations were distributed electronically to all panel members for review and confirmation. No recommendation was retained if substantive unresolved disagreement remained.

#### Limitations

Several limitations of this consensus process should be acknowledged. First, this document was developed using a structured narrative review rather than a formal systematic review, and formal risk-of-bias assessment or GRADE certainty ratings were not performed. Second, because the evidence base for FRα testing is rapidly evolving, some recommendations—particularly those related to timing of testing, reflex testing strategies, and implementation across provincial health systems—reflect expert consensus and practical Canadian considerations in addition to direct clinical trial evidence. Third, although consensus thresholds and iterative review were used to finalize recommendations, the process was not designed as a Delphi study. These limitations should be considered when interpreting the recommendations, which are intended to support practical implementation and equitable access rather than serve as a fully evidence-graded clinical practice guideline.

### 2.7. Patient Advocate Review

Patient representatives from Ovarian Cancer Canada were invited to provide input to ensure the recommendations aligned with patient priorities and addressed potential barriers to equitable access. Two one-hour-long sessions were held with 16 patient partners. Their insights informed revisions related to communication, access, and clarity of recommendations. Patient partners emphasized the importance of ongoing patient engagement as testing practices and policies evolve.

### 2.8. Use of Generative Artificial Intelligence

Generative artificial intelligence tools were not used in any component of the original consensus development process, including literature review, drafting, figure/table creation, or recommendation generation. All scientific and editorial work was performed by clinicians, scientists, and medical writers.

## 3. Results and Discussion

The recommendations from the expert working group are summarized in Table 1.

### 3.1. Testing Algorithm for FRα

Recommendation 1: FRα status should be established in patients with epithelial ovarian cancer who may become candidates for FRα-directed therapy, particularly those with non-mucinous histologies and recurrent or platinum-resistant disease.

FRα is a clinically actionable biomarker in epithelial ovarian cancer, as high FRα expression identifies patients who may benefit from FRα-directed therapy, including mirvetuximab soravtansine in the platinum-resistant setting [15,17]. However, FRα expression varies by histologic subtype, with the highest and most consistent expression in high-grade serous ovarian carcinoma, more variable expression in low-grade serous, endometrioid, and clear cell carcinomas, and uncommon expression in mucinous carcinoma [8,9].

Accordingly, FRα testing should be prioritized for patients most likely to become candidates for FRα-directed therapy, particularly those with recurrent or platinum-resistant non-mucinous EOC. Reflex testing at diagnosis may be considered for advanced-stage non-mucinous disease where resources allow, while selective testing is reasonable for mucinous carcinoma based on clinical context, diagnostic uncertainty, trial eligibility, local policy, or anticipated therapeutic access. Somatic testing guidance also recognizes FRα as a relevant actionable biomarker in recurrent ovarian cancer.

Recommendation 2: FRα testing results should be available no later than the time of platinum-resistant disease, when FRα-directed therapy becomes clinically actionable.

All panellists agreed that FRα results should be available by the time of platinum resistance. Reflex testing at diagnosis or first surgery may be considered when sufficient tissue is available, particularly for patients at higher risk of recurrence, as it may facilitate clinical trial enrolment, avoid delays at relapse, and use higher-quality diagnostic or surgical tissue [30,31]. However, universal reflex testing may increase upfront workload and cost, especially in lower-risk patients; only 30–50% of early-stage FIGO I–II HGSOCs recur, compared with >70% of advanced-stage FIGO III–IV disease, and approximately 70% of recurrent cases eventually become platinum-resistant [32,33].

Testing at first relapse is also reasonable and aligns with the current approved use of MIRV in platinum-resistant ovarian cancer [18,19,34,35]. This approach reduces upfront testing burden but may risk delays from archival tissue retrieval, centralized testing, assay turnaround time, or inadequate specimens. Therefore, timing should be guided by recurrence risk, tissue availability, laboratory capacity, reimbursement, and the need to ensure timely access to FRα-targeted therapy.

### 3.2. Pre-Analytical Considerations

Recommendation 3. Testing should be performed on tumour tissue fixed in 10% neutral buffered formalin for 6 to 48 h, as inadequate or prolonged fixation can compromise antigen preservation. Conventional cytology preparations are not validated for FRα testing; however, FFPE-processed cytology cell blocks may be considered where sufficient viable tumour is present and the specimen type has been appropriately validated by the testing laboratory.

Proper pre-analytical handling is essential to ensure accurate and reproducible FRα immunohistochemistry results [36,37]. FRα expression appears relatively stable across the disease course and generally concordant between archival and more recent tumour specimens, thus supporting the use of initial biopsy or primary debulking tissue as the preferred substrate when adequate material is available [14]. Re-biopsy should not be routine but may be warranted if archival tissue is unavailable, inadequate, exhausted, poorly fixed, equivocal, or previously tested with an unvalidated assay. Although re-biopsy may provide high-quality material, it can delay treatment; reflex testing at diagnosis may reduce this need and help preserve tissue.

Specimens should be handled carefully to avoid crush artifact and minimize ischemic time, both of which may impair staining quality [36,37]. FRα testing may be performed on resection specimens or core biopsies when sufficient tumour is present [14,37]. For patients diagnosed using ascites or pleural effusion specimens, FFPE-processed cell blocks may be acceptable if fixation, tumour cellularity, and assay performance have been locally validated; cytology smears and liquid-based preparations are not currently validated for FRα testing [37].

### 3.3. Analytical Considerations

Recommendation 4. To ensure clinical validity, biomarker testing should be performed by a licensed, accredited laboratory trained in FRα testing and reported by pathologists trained to interpret the specific assay used. Careful block selection is required to ensure adequate viable tumour and representative assessment.

To ensure clinical validity, FRα testing should be performed in laboratories trained and experienced in FRα immunohistochemistry and interpretation. Unlike DNA-based biomarkers, such as BRCA mutations or HRD scores, FRα is assessed at the protein level, primarily by IHC [38,39]. IHC is accessible, cost-efficient, and compatible with oncology turnaround times, but variation in antibody clones, staining platforms, scoring systems, and laboratory practices can affect reproducibility and patient eligibility [36].

Block selection should prioritize tissue with abundant viable invasive tumour, adequate fixation, and minimal necrosis, crush artifact, cautery artifact, or poor preservation. Areas with extensive necrosis, scant tumour cellularity, or predominantly benign/non-invasive tissue should be avoided. The selected block should be representative of tumour morphology and contain sufficient tumour for reliable scoring, with a minimum of 100 viable tumour cells recommended for interpretation. Samples with low tumour content, particularly <10%, may be suboptimal and should be interpreted with caution.

Interpretation should be performed by accredited pathologists with documented proficiency in FRα scoring to reduce misclassification and inappropriate treatment selection. Given potential intratumoral heterogeneity, assessment of additional blocks may be considered when staining is heterogeneous, tumour content is limited, results are equivocal, or morphology and staining appear discordant. These measures help reduce sampling-related variability and improve reliability of FRα interpretation.

Recommendation 5. Testing should be performed using a clinically validated assay. For mirvetuximab soravtansine eligibility, the currently validated threshold is FRα expression in ≥75% of viable tumour cells with ≥2+ membranous staining intensity using the Ventana FOLR1 RxDx assay.

**Figure 1:**
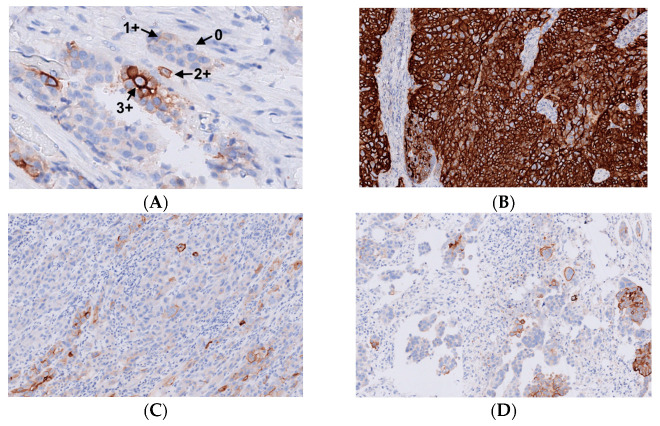
Representative FRα immunohistochemistry staining patterns in tubo-ovarian high-grade serous carcinoma. (**A**) Range of FOLR1 staining intensity from 0 to 3+. (**B**) High FRα expression, with ≥75% of viable tumour cells showing moderate-to-strong membranous staining, consistent with the currently validated Ventana PS2+ threshold for mirvetuximab soravtansine eligibility. (**C**) Low/focal FRα expression, with <25% of viable tumour cells showing moderate-to-strong membranous staining. (**D**) Negative or minimal FRα expression, with absent or only rare weak membranous staining. Images provided by Dr. Basile Tessier-Cloutier.

The Ventana FOLR1/FOLR1-2.1 RxDx Assay is currently the only Health Canada/FDA-approved companion diagnostic for mirvetuximab soravtansine [34,40,41] and was used in SORAYA and MIRASOL to define eligibility [15,17,42]. Results are reported as the percentage of viable tumour cells with membranous staining by intensity category, 0, 1+, 2+, or 3+; cytoplasmic staining is disregarded; see Figure 1 [37]. The currently validated FRα-positive threshold for mirvetuximab soravtansine eligibility is ≥75% of viable tumour cells with ≥2+ membranous staining, based on early-phase and pivotal studies showing greater benefit in patients with high FRα expression [42,43,44,45]. Although lower thresholds, including ≥50% or ≥25% at ≥2+, have been explored, they are not currently prospectively validated for this indication.

If the Ventana assay is unavailable, a laboratory-developed test may be used only if analytically validated against a clinically validated reference standard [41]. Alternative antibody clones and platforms have been investigated [46,47] but require careful validation. In the first QuIP^®^ interlaboratory FRα proficiency study, the Ventana FOLR1 RxDx assay had the highest success rate, 83%, whereas alternative clones such as BN3.2 and EPR20277 achieved substantially lower success rates, 22–25%, largely because of weaker staining intensity and lower concordance with reference values [48]. These findings reinforce the need for assay-specific validation, concordance around the ≥75% at ≥2+ threshold, appropriate controls, inter-/intra-observer reproducibility assessment, and participation in external quality assurance programs [41].

Recommendation 6. To minimize variability, all laboratories performing FRα testing must participate in external quality assurance and proficiency testing programs.

Participation in an external quality assurance program is also required to support ongoing proficiency and benchmarking across laboratories. Together, these measures ensure that alternative platforms achieve concordance sufficient to guide therapeutic decision making.

Internal quality assurance should include both positive and negative controls for each run and regular review of staining quality.

External quality assurance involves participation in the Canadian Association of Pathologists (CAP-ACP) or international External Quality Assessment schemes once available. Proficiency testing is recommended to include inter-laboratory slide exchanges and blinded scoring. Pathologist calibration can leverage dual reading during initiation and periodic concordance checks. These safeguards minimize false positives or negatives that impact patient access.

### 3.4. Post-Analytical Considerations

Recommendation 7. Timely FRα results should be incorporated into standardized synoptic pathology reports to streamline integration into management plans.

FRα immunohistochemistry results should be communicated through clear, standardized reporting to support consistent clinical interpretation. At minimum, each report must specify the assay used; the percentage of viable tumour cells at each staining intensity; the overall interpretation, including whether the tumour meets the ≥75% 2+ threshold for positivity; and relevant specimen information, such as tissue type and adequacy. Any assay-specific limitations that may affect interpretation should also be documented. The use of synoptic reporting templates aligned with College of American Pathologists (CAP) and American Society of Clinical Oncology biomarker frameworks is strongly recommended to promote uniformity and facilitate integration into clinical decision making [49].

Timely reporting is critical for patient management, particularly at recurrence when treatment decisions must be made quickly. FRα testing should adhere to clinical oncology turnaround expectations consistent with CAP guidelines [49], with results available within 7–10 working days for reflex testing performed at diagnosis and within 14 calendar days for testing initiated at relapse.

### 3.5. Patient Considerations

Recommendation 8: Clinicians must provide clear, accessible patient education regarding the role of FRα biomarker testing and engage in shared decision making regarding the timing of information disclosure.

As personalized treatments such as MIRV become more widely available in Canada, patient partners emphasized the importance of patients understanding the role of biomarker testing in determining whether—and to what extent—they are likely to benefit from precision therapies.

They encouraged clinicians to clearly communicate the purpose and implications of FRα and other biomarker testing so that patients are equipped to make treatment decisions aligned with their personal priorities. Patient partners further recommended that clinicians discuss patient preferences regarding the timing of information disclosure, such as receiving results immediately when available or only when results become clinically actionable.

Patient-informed consent is crucial for biomarker testing, and patient partners recommended that test results be communicated as early as possible in the care pathway, in accordance with individual patient preferences.

Recommendation 9: Access to psychological support services should be an integral component of the biomarker testing pathway to assist patients in navigating the emotional complexities of precision medicine.

Patient partners supported the availability of psychological support services throughout the biomarker testing process. Variability in patient preferences regarding the amount and timing of information highlights the need for flexible, patient-centred communication strategies.

### 3.6. Health-System Implementation Considerations

Recommendation 10: FRα testing should be reimbursed and available across Canada to reduce potential inequities in ovarian cancer treatment.

The introduction of FRα testing provides an important opportunity to individualize therapy for people with ovarian cancer; however, as seen with BRCA1/2, HRD, and PD-L1 testing, implementation barriers extend beyond technical considerations to broader issues of equity [30,50,51,52,53,54,55,56,57]. Precision oncology in Canada has historically shown uneven uptake across provinces, practice settings, and populations [52,58,59]. FRα testing faces similar risks, particularly in centres without Ventana platforms or validated alternatives. Geographic inequities further compound this problem: patients in rural or northern communities have historically had lower rates of molecular testing due to limited pathology resources, fewer specialists, and lack of reflex testing protocols [52].

Socioeconomic factors also influence diagnostic access. Evidence shows that women from lower-income, rural, Indigenous, and racialized communities experience reduced uptake of genetic and molecular testing, even within a universal healthcare system [52,53,55,56,60]. Out-of-pocket costs may arise before provincial reimbursement is established, and structural barriers—including travel distance, language obstacles, and mistrust of medical systems—further impede access to FRα testing and subsequent therapy.

To avoid reproducing these inequities, coordinated system-level strategies are required. Reflex FRα testing, centralized reimbursement strategies, integrated laboratory networks, and active partnerships with underserved communities are critical to ensuring timely and equitable access. Without such safeguards, FRα testing and FRα-directed therapies risk becoming accessible only to a subset of Canadians, reinforcing disparities observed in previous biomarker programs.

## 4. Perspectives of Patient Partners

A total of 16 patient partners provided feedback on the consensus statement following a review of FRα testing and the evidence for MIRV. They were enthusiastic about the integration of FRα testing into standard care, noting that expanded testing would increase access to treatment options. They also endorsed the recommendations outlined in this document that FRα testing be made available to all patients with EOC to reduce inequities in ovarian cancer care and recommended that FRα testing occur at diagnosis.

During the initial implementation phase, when laboratory capacity and infrastructure may temporarily limit access, patient partners recommended prioritizing patients with recurrent disease or platinum-resistant cancer who may derive immediate clinical benefit.

## 5. Future Directions and Conclusions

As FRα testing becomes integrated into routine EOC care, its successful implementation will depend not only on technical accuracy and laboratory readiness but also on coordinated national strategies that support equitable access across all regions of Canada. Continued advances in FRα-targeted therapeutics, including next-generation antibody–drug conjugates, immunotherapies, and imaging modalities, are likely to broaden the clinical relevance of FRα testing beyond platinum-resistant disease. Ongoing research will be essential to refine scoring thresholds, evaluate testing at earlier disease stages, and understand the biomarker’s role in emerging therapeutic combinations.

Sustained collaboration among clinicians, pathologists, provincial laboratories, policymakers, patient advocates, and regulatory agencies will be critical to ensure that diagnostic and therapeutic innovations progress in parallel. As precision oncology continues to evolve, FRα testing offers an opportunity to establish a scalable model for biomarker implementation—one that prioritizes standardization, quality assurance, health-system coordination, and equity.

Ultimately, the incorporation of FRα testing into the Canadian ovarian cancer landscape represents an important step toward more personalized and effective care. With harmonized national infrastructure and proactive policy alignment, Canada can ensure that all patients who may benefit from FRα-directed therapies are identified promptly and treated without delay, setting the stage for continued improvements in outcomes for people facing this devastating disease.

## Figures and Tables

**Table 1:** Summary of FRα testing recommendations for patients with epithelial ovarian cancer.

Consensus Statements
3.1 Testing Algorithm for FRα
FRα status should be established in patients with epithelial ovarian cancer who may become candidates for FRα-directed therapy, particularly those with non-mucinous histologies and recurrent or platinum-resistant disease.
2. FRα testing results should be available no later than the time of platinum-resistant disease, when FRα-directed therapy becomes clinically actionable.
3.2 Pre-Analytical Considerations
3. Testing should be performed on tumour tissue fixed in 10% neutral buffered formalin for 6 to 48 h, as inadequate or prolonged fixation can compromise antigen preservation. Conventional cytology preparations are not validated for FRα testing; however, FFPE-processed cytology cell blocks may be considered where sufficient viable tumour is present and the specimen type has been appropriately validated by the testing laboratory.
3.3 Analytical Considerations
4. To ensure clinical validity, biomarker testing should be performed by a licensed, accredited laboratory trained in FRα testing and reported by pathologists trained to interpret the specific assay used. Careful block selection is required to ensure adequate viable tumour and representative assessment.
5. Testing should be performed using a clinically validated assay. For mirvetuximab soravtansine eligibility, the currently validated threshold is FRα expression in ≥75% of viable tumour cells with ≥2+ membranous staining intensity using the Ventana FOLR1 RxDx assay.
6. To minimize variability, all laboratories performing FRα testing must participate in external quality assurance and proficiency testing programs.
3.4 Post-Analytical Considerations
7. FRα results should be incorporated into standardized synoptic pathology reports to streamline integration into management plans.
3.5 Patient Considerations
8. Clinicians must provide clear, accessible patient education regarding the role of FRα biomarker testing and engage in shared decision making regarding the timing of information disclosure. 9. Access to psychological support services should be an integral component of the biomarker testing pathway to assist patients in navigating the emotional complexities of precision medicine.
3.6 Health-System Implementation Considerations
10. FRα testing should be reimbursed and available across Canada to reduce potential inequities in ovarian cancer treatment.

## Data Availability

No new data were created or analyzed in this study. Data sharing is not applicable to this article.

## Abbreviations

The following abbreviations are used in this manuscript:

BiTE	Bispecific T-cell engagers
CAP	College of American Pathologists
EOC	Epithelial ovarian cancer
FDA	Food and Drug Administration
GOC	Gynecologic Oncology of Canada
HGS	High-grade serous
HRD	Homologous recombination deficiency
IHC	Immunohistochemistry
MIRV	Mirvetuximab soravtansine
PARP	Poly-(ADP-ribose)-polymerase
